# Oligomerization of the Polycystin-2 C-terminal Tail and Effects on Its Ca^2+^-binding Properties*

**DOI:** 10.1074/jbc.M115.641803

**Published:** 2015-02-25

**Authors:** Yifei Yang, Camille Keeler, Ivana Y. Kuo, Elias J. Lolis, Barbara E. Ehrlich, Michael E. Hodsdon

**Affiliations:** From the Departments of ‡ Laboratory Medicine,; § Pharmacology, and; ¶ Cellular and Molecular Physiology, School of Medicine, Yale University, New Haven, Connecticut 06520

**Keywords:** Calcium, Calcium Intracellular Release, Calcium-binding Protein, Isothermal Titration Calorimetry (ITC), Nuclear Magnetic Resonance (NMR), EF-hand Proteins, Polycystic Kidney Disease

## Abstract

Polycystin-2 (PC2) belongs to the transient receptor potential (TRP) family and forms a Ca^2+^-regulated channel. The C-terminal cytoplasmic tail of human PC2 (HPC2 Cterm) is important for PC2 channel assembly and regulation. In this study, we characterized the oligomeric states and Ca^2+^-binding profiles in the C-terminal tail using biophysical approaches. Specifically, we determined that HPC2 Cterm forms a trimer in solution with and without Ca^2+^ bound, although TRP channels are believed to be tetramers. We found that there is only one Ca^2+^-binding site in the HPC2 Cterm, located within its EF-hand domain. However, the Ca^2+^ binding affinity of the HPC2 Cterm trimer is greatly enhanced relative to the intrinsic binding affinity of the isolated EF-hand domain. We also employed the sea urchin PC2 (SUPC2) as a model for biophysical and structural characterization. The sea urchin C-terminal construct (SUPC2 Ccore) also forms trimers in solution, independent of Ca^2+^ binding. In contrast to the human PC2, the SUPC2 Ccore contains two cooperative Ca^2+^-binding sites within its EF-hand domain. Consequently, trimerization does not further improve the affinity of Ca^2+^ binding in the SUPC2 Ccore relative to the isolated EF-hand domain. Using NMR, we localized the Ca^2+^-binding sites in the SUPC2 Ccore and characterized the conformational changes in its EF-hand domain due to trimer formation. Our study provides a structural basis for understanding the Ca^2+^-dependent regulation of the PC2 channel by its cytosolic C-terminal domain. The improved methodology also serves as a good strategy to characterize other Ca^2+^-binding proteins.

## Teaser

**Background:** The C-terminal tail of polycystin-2 (PC2 Cterm) is essential for channel assembly and regulation.

**Results:** Both human and sea urchin PC2 Cterm form trimers and contain EF-hand domains that bind to Ca^2+^.

**Conclusion:** Oligomerization affects Ca^2+^-binding profiles differently in human and sea urchin PC2.

**Significance:** Characterization of the PC2 Cterm aids the understanding of PC2 channel regulation.

## Introduction

Autosomal dominant polycystic kidney disease is a prevalent heterozygous genetic disorder that leads to the development of renal cysts and ultimately kidney failure (1, 2). Autosomal dominant polycystic kidney disease has two disease loci in humans, *pkd1* and *pkd2*, which encode polycystin-1 and polycystin-2 (PC2)^5^ (3). PC2, a member of the transient receptor potential (TRP) channel family, has six putative transmembrane helices and a pore-forming loop. Unlike most TRP proteins, PC2 predominantly localizes to the endoplasmic reticulum, where it contributes to intracellular Ca^2+^ flux from the endoplasmic reticulum (4). PC2 is a Ca^2+^-permeable channel, and its open probability is regulated by cytoplasmic Ca^2+^ levels. The open probability of the channel follows a bell-shaped curve depending on Ca^2+^ concentration (5). At low Ca^2+^ levels, an increase in Ca^2+^ concentration results in higher open probability, whereas a further increase in Ca^2+^ concentration reduces the channel open probability. It is unclear exactly how the PC2 channel transitions between these two modes of Ca^2+^ regulation. The C-terminal tail of human PC2 (HPC2 Cterm) is known to be important for the regulation and assembly of the PC2 channel (4, 5, 7). It contains two identified domains, a Ca^2+^-binding EF-hand and a coiled-coil domain shown to oligomerize, which have been studied separately (7–10). However, how the Ca^2+^-binding properties of full-length C-terminal tail are affected by its oligomeric state has not been rigorously studied. Their interactions are believed to contribute to the more complex states of PC2 channel regulation. The coiled-coil domain (amino acids 830–872 of human PC2) is involved in the assembly of PC2 channel as well as in hetero-oligomerization with other proteins (11–13). Intriguingly, the crystal structure of the isolated human PC2 coiled-coil domain suggests that it forms a trimer, although TRP channels are generally tetramers (10). Whether the same trimer structure exists with the full-length HPC2 Cterm, which includes both the EF-hand and coiled-coil domain, is not known. Because trimer formation is highly unusual for a TRP channel, it is important to verify this by solution-based biophysical approaches. However, studying the HPC2 Cterm in solution has proved challenging, largely because the protein is prone to forming aggregates in solution without Ca^2+^ (14). When analyzed with Ca^2+^, the non-spherical shape of the protein complex further complicates the characterization (7, 14, 15). Thus, a detailed biophysical characterization of HPC2 Cterm requires an optimized solution in which the distribution of oligomeric states is specific and stable.

**FIGURE 1.**
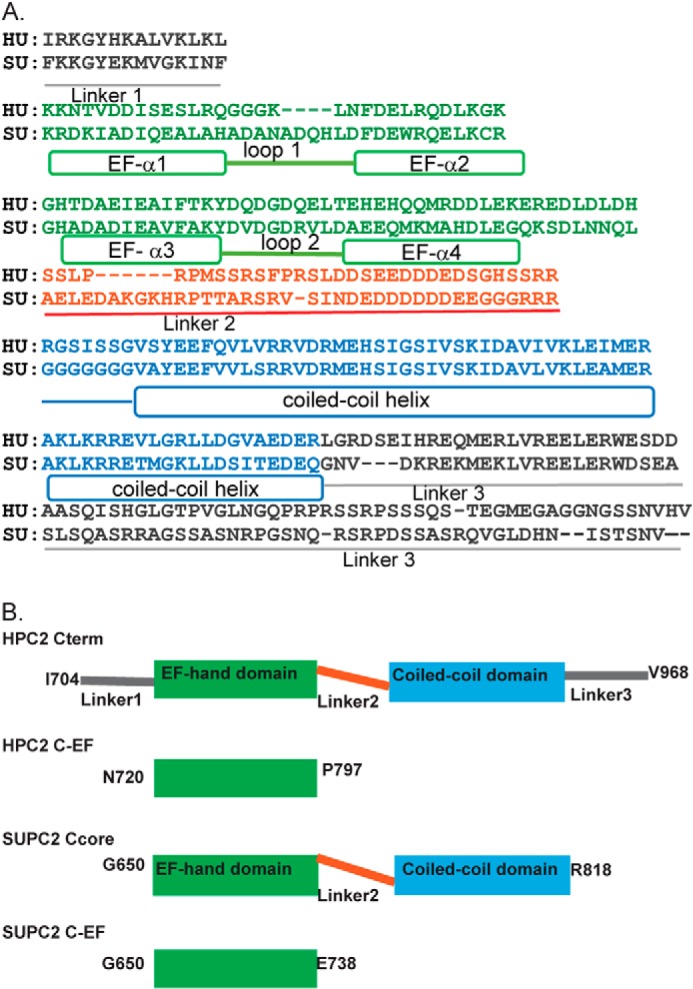
**The PC2 C-terminal domains in human and sea urchin PC2.** *A*, alignment of the C-terminal domain sequence of human and sea urchin PC2 homologues. *B*, different domains are included in the construct design. The constructs are positioned based on sequence alignment results. The *residue numbers* mark the start and finish residues of each construct, in the context of full-length human and sea urchin PC2 proteins, respectively. Therefore, the human and sea urchin constructs are numbered differently.

Characterizing the Ca^2+^-binding elements postulated to reside within the PC2 C-terminal tail is crucial for understanding the mechanism of channel regulation. Based on the bell-shaped response of open probability to increasing Ca^2+^ concentration, it can be hypothesized that separate Ca^2+^-binding sites are responsible for different sides of the bell-shaped curve (4, 5, 7, 9, 16). The EF-hand domain (amino acids 719–798 of human PC2) contains one Ca^2+^-binding site, which is important for the Ca^2+^-dependent regulation of PC2 channel (7, 9). However, the isolated EF-hand domain binds Ca^2+^ very weakly, with a *K_D_* of 461 μm, outside the physiologic range of cytosolic Ca^2+^ concentrations (17). Therefore, it was speculated that there could be additional Ca^2+^-response elements located in the C-terminal domain outside the EF-hand region (7). Moreover, a series of acidic residues located in the loop region (linker 2-L2) (Fig. 1*A*) connecting the EF-hand and coiled-coil domain share a sequence similar to the Ca^2+^-bowl structure found in the BK (big potassium) channel (18, 19). It was important to determine the Ca^2+^-binding profiles of HPC2 Cterm because this would address the question of whether there are additional C-terminal Ca^2+^-binding sites outside the EF-hand domain and if the Ca^2+^-binding affinity of HPC2 Cterm is within the physiologic range (nanomolar to low micromolar). However, the Ca^2+^-binding profile of the HPC2 Cterm was not fully determined in previous studies using isothermal titration calorimetry (ITC) due to the presence of residual Ca^2+^ and protein aggregates (7).

The goal of our study is to define the oligomeric state of PC2 C-terminal tail in solution, to characterize its Ca^2+^-binding profile including affinity and stoichiometry, and to map its Ca^2+^-response elements. The C-terminal domain sequence of PC2 is highly conserved across different species. To overcome the inherent issues of protein degradation and aggregation associated with the HPC2 Cterm, we used the sea urchin PC2 (SUPC2) orthologue to study the conformational changes and domain-domain interactions within the PC2 C-terminal tail. The human and sea urchin PC2 C-terminal domains share 52% sequence identity and the same domain topology (Fig. 1*A*). For our biophysical studies, a newly developed ITC approach (17) enabled us to quantify both the residual Ca^2+^ concentration and the binding properties of the high affinity Ca^2+^ binding sites, parameters that were previously difficult to measure. We also established a buffer system to obtain a stable distribution of the oligomeric states and to prevent the aggregation that complicated previous solution state studies. We used a light scattering technique that can determine the absolute molar mass of the oligomer complex. Using NMR, we mapped the Ca^2+^-binding sites and described the Ca^2+^-dependent conformational changes in the sea urchin C-terminal tail. Our study, which focuses on the oligomeric states and the Ca^2+^-binding profiles of the PC2 C-terminal domains, provides insight into how the PC2 channel could be regulated by its cytosolic C-terminal domain. Our knowledge also leads to an improved understanding of the functional role of PC2 in regulating intracellular Ca^2+^ signaling.

## EXPERIMENTAL PROCEDURES

### 

#### 

##### Protein Constructs and Cloning

The following constructs were amplified using PCR from human PC2 cDNA or sea urchin PC2 cDNA and cloned into pET-28(a+) (Novagen) with an N-terminal His tag: HPC2 Cterm (human PC2, I704-V968); HPC2 C-EF (human PC2, N720-P797); SUPC2 C-EF (sea urchin PC2, G655-E738). The SUPC2 Ccore construct (sea urchin PC2, G650-R818) was synthesized by Genscript with codon optimization according to its original amino acid sequence. Two site mutations (KINF → KIDD and NNQL → HNEM), located in the N and C termini were introduced to prevent proteolytic degradation. All constructs were cloned into pET-28(a+) (Novagen) with an N-terminal His tag. The detailed domain topology of the constructs is described in the legend to Fig. 1*B*.

##### Recombinant Protein Expression and Purification

HPC2 Cterm, SUPC2 Ccore, and SUPC2 C-EF constructs were expressed and purified by nickel affinity chromatography as described previously (9) with minor modifications for each construct. The eluted fractions from the nickel affinity column were desalted and further purified by gel filtration chromatography (GE Healthcare). The sea urchin constructs were eluted with buffer containing 150 mm KCl, 25 mm Tris, 1 mm tris(2-carboxyethyl)phosphine (TCEP), and 20 mm CaCl_2_, at a pH of 7.4 (Buffer A). TCEP was used to maintain the reduced form of Cys residues in the SUPC2 Ccore and SUPC2 C-EF proteins. The HPC2 Cterm construct was eluted with buffer containing 150 mm KCl, 25 mm Tris, 20 mm CaCl_2_, and 20 mm imidazole, with a pH of 7.4 (Buffer B). The addition of 20 mm imidazole was used to maintain a monodispersed peak during the elution and to prevent the HPC2 Cterm from forming large nonspecific complexes and precipitating in solution. Without the presence of 20 mm imidazole, the HPC2 Cterm protein construct was found to form aggregates in solution that interfered with the biophysical analysis. In addition to imidazole, we also tried other solvent components that were known to improve protein solubility and stability, such as 10% glycerol and 20–100 mm sucrose. Of all of the solvents, imidazole was shown to be most effective in improving the solubility of the HPC2 Cterm protein and is compatible with the solution system for our biophysical analysis.

##### Size Exclusion Chromatography-Multiangle Light Scattering (SEC-MALS) Experiment to Determine Molar Mass

SEC was used to monitor the distribution of oligomeric states in multiple constructs in both the Ca^2+^-bound (holo) and Ca^2+^-free (apo) states. To ensure that all possible Ca^2+^-binding sites were saturated during the holo state analysis, 20 mm CaCl_2_ was added to the buffer. The analysis under apo conditions was achieved by using buffer containing additional 1 mm EDTA to strip away any residual Ca^2+^ bound to the protein. The protein sample was injected onto a Superdex200 10/30 GL column attached to an AKTA FPLC (GE Healthcare) at 4 °C. The elution was monitored by UV absorbance at 280 nm. The HPC2 Cterm and SUPC2 Ccore constructs were analyzed under holo and apo conditions, respectively. The peak fractions were analyzed by SDS-PAGE and mass spectrometry (MS) to confirm that the protein samples still remained intact after analysis.

Coupled with SEC, MALS analyses were conducted to determine the molar mass of the elution peaks for each construct in both holo and apo states. The light scattering data were collected as described previously (20) with customized running buffers for different states. The SEC-UV/LS/RI system was equilibrated in customized buffer at a flow rate of 1.0 ml/min. The weight average molar mass was determined by averaging the molar mass measurement across the entire elution profiles in intervals of 1 s from static LS measurement using ASTRA software as described previously (21).

##### ITC to Characterize Ca^2+^ Binding

The Ca^2+^-binding properties of different PC2 constructs were characterized by measuring the heats generated by Ca^2+^ binding. Calorimetry experiments were performed on a Nano ITC Low Volume instrument (TA Instruments) and a VP-ITC instrument (GE Healthcare). Purified protein samples were placed in the sample cells, and ligand CaCl_2_ was titrated in the cell through the titration syringe. To prepare ITC samples for HPC2 Cterm, several steps of buffer exchange were performed to remove excessive CaCl_2_. The final ITC samples were prepared in buffer containing 150 mm KCl, 25 mm Tris, and 20 mm imidazole, pH 7.4, buffer. Similar steps of buffer exchange were applied to the SUPC2 Ccore and the SUPC2 C-EF ITC samples, and the samples were prepared in 150 mm KCl, 25 mm Tris, and 1 mm TCEP, pH 7.4, buffer. The protein concentrations of the HPC2 Cterm and the SUPC2 Ccore were determined by UV absorbance in denaturing conditions at 280 nm. The SUPC2 C-EF protein concentration was determined by amino acid analysis.

Initial ITC experiments without Ca^2+^ chelators revealed residual Ca^2+^ in the samples even after performing the series of buffer exchanges. If the *K_D_* for the Ca^2+^-binding affinity of the protein lies in a low micromolar range, even a micromolar level of residual Ca^2+^ in the sample will render a significant proportion of protein unavailable for ITC characterization. To account for the micromolar level of residual Ca^2+^ bound to the protein sample and more accurately determine binding parameters, a new approach was developed to model ITC data using an alternative mathematical framework (17). The residual ligand is accounted by the addition of a well characterized ligand chelator (22, 23). In the new approach, the ITC experiments were conducted by pairing each Ca^2+^-protein titration experiment with another matching Ca^2+^ chelator-protein experiment. When protein samples were prepared, half of the sample was used for a regular Ca^2+^ titration experiment, and Ca^2+^ chelator (EDTA or 5,5′-dimethyl-BAPTA) was added to the other half for subsequent ITC experiments. In addition, separate ITC experiments and Ca^2+^-selective electrode measurements were performed to determine the binding thermodynamic parameters of chelators (EDTA or 5,5′-dimethyl-BAPTA) in each buffer system.

ITC baseline corrections were performed using the NanoAnalyze software (TA Instruments). Data were exported and converted to the correct units. The data set was then analyzed in Mathematica (Wolfram Research) by scripts previously developed and validated in the laboratory, available upon request (17). In the ITC analysis, three different predefined binding models are used: the *identical binding sites model*, the *two independent binding sites model*, and the *two cooperative binding sites model* (24–26). In all three binding models, the concentration of the macromolecule is defined as the protein monomer concentration. Therefore, the number of binding sites (binding stoichiometry) describes the number of binding sites within each monomer. The identical binding sites model describes the binding interaction with any number of identical binding sites, and the binding sites are independent of each other. The two independent binding sites model was defined as two sets of binding sites with different binding affinities (*K_D_*) and changes in enthalpy (Δ*H*), with sites acting independently of one another. In the two cooperative binding sites model, the occupation of one binding site affects the binding affinity of the other site by a factor defined as the cooperativity coefficient (*c*). The details regarding the definition of these binding models and the explicit forms of the isotherm equations are provided upon request.

For the HPC2 Cterm, the thermodynamic parameters of the ligand protein binding interaction were determined by fitting both binding isotherms simultaneously to the identical binding sites model. In order to limit the degrees of freedom for the binding model, the Ca^2+^ chelator parameters were set as prefixed values derived from separate Ca^2+^-binding ITC experiments during the fitting process. The best fit values for the thermodynamic parameters of the binding model were obtained by a numerical minimization of the residual sum of squares. The confidence intervals for each parameter were determined by plotting the residual sum of squares χ^2^ space around its best fit value, and the intervals that define the 95% critical values were reported as the 95% confidence interval.

For the SUPC2 C-EF and the SUPC2 Ccore, the ITC data were fitted with three different binding models (identical binding sites model, two independent binding sites model, and two cooperative binding sites model), and the fitting results from the three models were compared using statistical analysis (nested F-tests). The binding model that best describes the ITC data is the two cooperative binding sites model, with the cooperativity coefficient *c*. However, this model has more degrees of freedom, and the parameters were found to be interdependent during the fitting process. Notably, the binding parameters for the weaker binding site (*K_D_*_2_ and Δ*H*2) were not as well restrained as those for the tighter binding site (*K_D_*_1_ and Δ*H*1). Due to the inherent level of signal to noise in the ITC experiments, it was impossible to completely define the confidence intervals for the co-dependent binding parameters. The upper limits of the confidence intervals for the second site binding parameters *K_D_*_2_ and Δ*H*2 were not well defined if *c* was allowed to float. When *c* was fixed as the best fitted values, the confidence intervals of *K_D_*_2_ and Δ*H*2 could be better defined.

##### NMR Spectroscopy to Characterize Structural Changes under Different Ca^2+^ Levels

NMR experiments were performed at 25 °C on a Varian INOVA 600-MHz spectrometer as described previously (9). The NMR experiments were collected on ^15^N- and ^13^C-labeled 1.0 mm SUPC2 Ccore samples in both the holo state (20 mm CaCl_2_) and the apo state (1 mm EDTA) at pH 7.4 with 2 mm Tris-*d*_11_, 150 mm KCl, 1 mm TCEP, a protease inhibitor mixture (Roche Applied Science), and 5% (v/v) D_2_O, with 5 mm sodium azide added as a preservative.

The backbone assignments for the residues located in the EF-hand region of the SUPC2 Ccore under holo conditions were determined by manual analysis of two-dimensional heteronuclear single quantum coherence (HSQC) and a series of three-dimensional triple-resonance HNCACB, HNCO, HN(CA)CO, HNCA, and HN(CO)CA experiments with TROSY enhancement in SPARKY (UCSF) (27). Proton chemical shifts were referenced indirectly to 3-(trimethylsilyl)propionic-2,2,3,3-*d*_4_ acid at ^1^H 0.00 ppm, with indirect dimensions referenced based on their relative gyromagnetic ratios.

##### Comparing Chemical Shifts between SUPC2 Ccore and SUPC2 C-EF

The chemical shift values of the backbone residues in the SUPC2 Ccore EF-hand region and SUPC2 C-EF were used to calculate the chemical shift perturbation between two constructs. For each set of nuclei, the residue-specific perturbation (Δδ) was derived by subtracting the SUPC2 C-EF chemical shift values (δ_SUPC2C-EF_) from the corresponding SUPC2 Ccore chemical shift values (δ_SUPC2Ccore_): Δδ = δ_SUPC2Ccore_ − δ_SUPC2C-EF_.

To include all of the assigned backbone nuclei (^1^H^N^, ^15^N, CO, Cα, Cβ) in the assessment of chemical shift perturbation, the weighted average of chemical shift changes per residue Δδ_all nuclei_ was calculated using the formula,

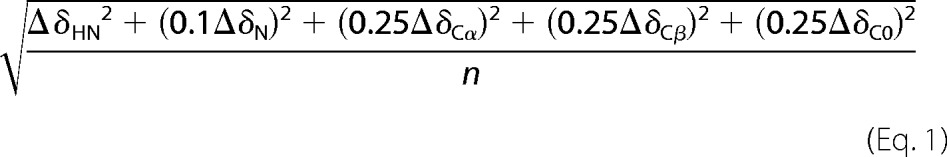
 The formula includes the chemical shift differences of all of the assigned nuclei (28). The scaling factors 0.1 and 0.25 represent the approximate gyromagnetic ratios of ^15^N nucleus to ^1^H and ^13^C nucleus to ^1^H, respectively (29, 30). The averaging factor *n* is the number of assigned nuclei for each backbone residue. There are altogether five nuclei used for the comparison of chemical shift perturbations, with some residues having certain nuclei unassigned or unavailable. The weighted averages of chemical shift differences for each residue were plotted along their primary sequence and along the corresponding secondary structure. The weighted averages of the chemical shift differences were also mapped to the known structure of the SUPC2 C-EF protein (Protein Data Bank code 2MHH) using PyMOL. The color of each residue is based on the level of chemical shift perturbation and plotted based on the “blue_white_green” color scheme, in which green indicates high chemical shift perturbation and blue indicates low chemical shift perturbation level. The unassigned residues are shown in gray.

## RESULTS

### 

#### 

##### Protease-resistant Mutants Allow for Biophysical Study

Four different human and sea urchin PC2 C-terminal constructs were selected for biophysical characterization (Fig. 1*A*). All of the constructs were tested and found to be stable at 25 °C for 24 h, as required for most of the biophysical experiments. However, NMR experiments require longer periods of protein stability, up to several days at 25 °C. The original wild-type SUPC2 Ccore construct, which includes the EF-hand domain and the coiled-coil domain (Fig. 1), are susceptible to proteolytic degradation over this time frame. Mutations were introduced to render the SUPC2 Ccore construct protease-resistant. The design of these mutations was guided by sequence analysis of the cleaved product. The mutated SUPC2 Ccore was stable at 25 °C for up to 20 days and was used in all subsequent studies. Conversely, the HPC2 Cterm was not stable for long periods of time (over 48 h) at room temperature, rendering it unsuitable for study using NMR.

##### Addition of Imidazole Prevents Aggregation of HPC2 Cterm in Solution

The HPC2 Cterm construct was found to aggregate at micromolar concentrations in buffer containing 25 mm Tris, 150 mm KCl, pH 7.4, rendering biophysical analysis difficult. After testing different buffer solutions, we found that the addition of 20 mm imidazole results in a monodispersed peak when the HPC2 Cterm sample was analyzed using SEC-UV analysis. Compared with several other stabilizing solvents, 20 mm imidazole was found to be most effective in stabilizing the HPC2 Cterm in solution and in preventing aggregation. The improved solubility and stability is postulated to result from a combination of improved hydration shell around the native protein and decreased nonspecific hydrophobic interactions between the disordered regions of the protein (31, 32). To prevent aggregation during the biophysical analyses, imidazole was added in the buffer solutions of all subsequent SEC-MALS and ITC experiments studying the HPC2 Cterm. This buffer modification allowed us to overcome the previous technical barriers that prevented the biophysical analysis of the HPC2 Cterm complex.

##### HPC2 Cterm and SUPC2 Ccore Form Trimers in Solution in Both the Apo and Holo States

**FIGURE 2.**
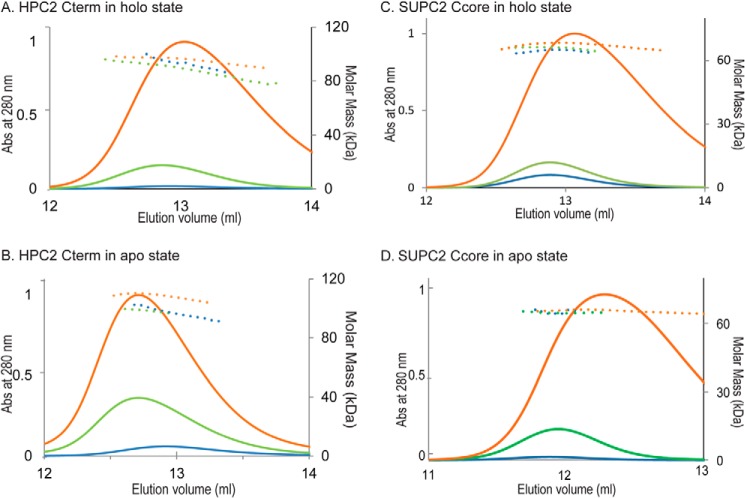
**SEC-MALS results of HPC2 Cterm and SUPC2 Ccore in Ca^2+^-bound holo and Ca^2+^-free apo states.** *A*, three different amounts of HPC2 Cterm protein were analyzed by Superdex 200 SEC-UV/LS/RI in Ca^2+^-saturating buffer (20 mm CaCl_2_). The *UV curves* indicate the elution peaks of the protein samples analyzed, and the *dotted lines* indicate the calculated molar mass across the elution peaks. *Orange*, 1.16-mg injection; *green*, 155-μg injection; *blue*, 31.0-μg injection. *B*, three different amounts of HPC 2Cterm protein were analyzed by Superdex 200 SEC-UV/LS/RI in Ca^2+^-free buffer (no added CaCl_2_, 1 mm EDTA). *Orange*, 2.36-mg injection; *green*, 650-μg injection; *blue*, 260-μg injection. *C*, three different amounts of SUPC2 Ccore protein were analyzed by Superdex 200 SEC-UV/LS/RI in Ca^2+^-saturating buffer (20 mm CaCl_2_). The *UV curves* indicate the elution peaks of the protein samples analyzed, and the *dotted lines* indicate the calculated molar mass across the elution peaks. *Orange*, 1.35-mg injection; *green*, 150-μg injection; *blue*, 15.0-μg injection. *D*, three different amounts of SUPC2 Ccore protein were analyzed by Superdex 200 SEC-UV/LS/RI in Ca^2+^-free buffer (no added CaCl_2_, 1 mm EDTA). *Orange*, 1.35-mg injection; *green*, 150-μg injection; *blue*, 75.2 μg injection.

The addition of imidazole allows for measurement of the absolute molar mass of the HPC2 Cterm oligomer. Based on its molar mass, the oligomeric states of the HPC2 Cterm in solution were then determined under both apo (1 mm EDTA) and holo (20 mm CaCl_2_) conditions. Under each condition, different quantities of the HPC2 Cterm were analyzed to examine whether its oligomeric states are dependent on protein concentration. Under both conditions, similar ranges of molecular weight, determined by static light scattering, were observed for all three ranges of eluting concentrations (Fig. 2, *A* and *B*). The averaged values of molar mass were very similar for both apo and holo conditions, 96.5 kDa (apo) and 95.0 kDa (holo) (Table 1), respectively. With a monomeric mass of 32.2 kDa, the HPC2 Cterm forms a trimer independent of protein concentration over the tested range, with or without bound Ca^2+^. Based on parallel analyses of the SUPC2 Ccore, the protein appears to have a similar range of molar mass across three concentrations under both apo and holo conditions (Fig. 2, *C* and *D*). Because the monomer of the SUPC2 Ccore protein is predicted to have a mass of 21.3 kDa, the averaged molar mass for the two states obtained by static light scattering, 65.3 kDa (apo) and 67.0 kDa (holo), also suggests that the SUPC2 Ccore forms a trimer in solution, independent of its concentration in the examined range and the presence of Ca^2+^.

**TABLE 1:** **The oligomeric states and hydrodynamic radii of different PC2 protein constructs** SEC-MALS and dynamic light scattering (DLS) results of the various PC2 protein constructs determined their oligomeric states and hydrodynamic radius (*r_h_*) in Ca^2+^-free apo and Ca^2+^-bound holo states.

Protein sample (Ca^2+^ states)	r*_h_*	Average molar mass (range)	Monomer mass	Oligomeric states
	*nm*	*kDa*	*kDa*	
HPC2 Cterm	–	95	32.2	Trimer
Holo state		(85–105)		
HPC2 Cterm	6.12 ± 0.17	96.5	32.2	Trimer
Apo state		(85–103)		
SUPC2 Ccore	4.41 ± 0.15	67	21.6	Trimer
Holo state				
SUPC2 Ccore	5.30 ± 0.17	65.3	21.6	Trimer
Apo state				
HPC2 C-EF	–	11.2	11.4	Monomer
Holo state		(10.7–11.5)		
SUPC2 C-EF	–	12.1	12.8	Monomer
Holo state		(11.5–12.5)		

We also measured the hydrodynamic radius and shape of the molecule by coupling dynamic light scattering with SEC-MALS. Dynamic light scattering data show that the HPC2 Cterm trimer is highly non-spherical, with a hydrodynamic radius (*r_h_*) of 6.12 ± 0.17 nm. Under apo and holo conditions, the elution volumes of the peaks for the construct are very close, indicating a similar non-spherical and elongated molecular shape in both cases. For the SUPC2 Ccore, in holo form, the measured *r_h_* is 4.41 ± 0.15 nm. In its apo state, the *r_h_* is slightly larger, 5.30 ± 0.17 nm (Table 1). Such a shift in hydrodynamic radius was also confirmed by the difference in elution volumes, 12.3 ml (apo) and 13.1 ml (holo). Irrespective of shape, the calculated molar masses from the static light scattering measurements were very similar, indicating that both the HPC2 Cterm and the SUPC2 Ccore form a trimer in solution in both Ca^2+^-bound and Ca^2+^-free states.

##### HPC2 Cterm Contains One Ca^2+^-binding Site with Significantly Higher Ca^2+^-binding Affinity Relative to HPC2 C-EF

**FIGURE 3.**
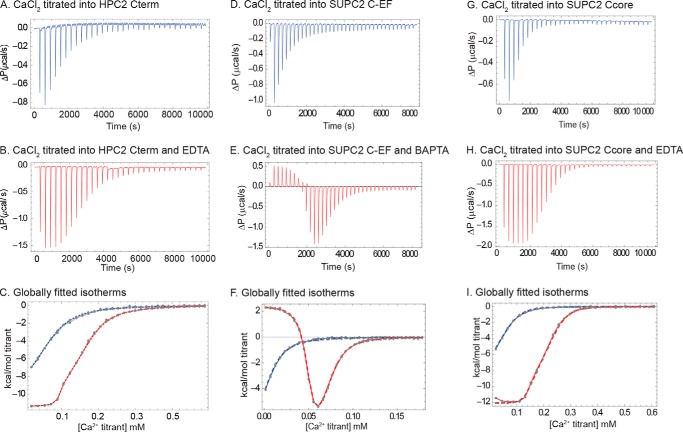
**ITC measurement of PC2 C-terminal constructs and Ca^2+^ binding interaction.** *A*, raw heat measurement of 1.96 mm CaCl_2_ titrated into 100 μm HPC2 Cterm protein in pH 7.4, 25 mm Tris, 150 mm KCl, 20 mm imidazole buffer at 25 °C, using 32 injections of 1.49 μl/injection. *B*, raw heat measurement of 1.96 mm CaCl_2_ titrated into 98.5 μm HPC2 Cterm protein and 115 μm EDTA in pH 7.4, 25 mm Tris, 150 mm KCl, 20 mm imidazole buffer at 25 °C, using 32 injections of 1.49 μl/injection. *C*, simultaneously fitted, baseline-corrected isotherms of CaCl_2_ into HPC2 Cterm only (*blue trace*) and protein with EDTA (*red trace*) ITC experiment. *D*, raw heat measurement of 1.00 mm CaCl_2_ titrated to 15.2 μm SUPC2 C-EF protein in pH 7.4, 25 mm Tris, 150 mm KCl, 1 mm TCEP buffer at 25 °C, using 36 injections of 8 μl/injection. *E*, raw heat measurement of 1.00 mm CaCl_2_ titrated into 13.3 μm SUPC2 C-EF protein and 88 μm 5,5′-dimethyl-BAPTA in pH 7.4, 25 mm Tris, 150 mm KCl, 1 mm TCEP buffer at 25 °C, using 36 injections of 8 μl/injection. *F*, simultaneously fitted, baseline-corrected isotherms of SUPC2 C-EF only (*blue trace*) and SUPC2 C-EF with 5,5′-dimethyl-BAPTA (*red trace*) ITC experiment. *G*, raw heat measurement of 2.44 mm CaCl_2_ titrated into 88 μm SUPC2 Ccore protein in pH 7.4, 25 mm Tris, 150 mm KCl, 1 mm TCEP buffer at 25 °C, using 32 injections of 1.49 μl/injection. *H*, raw heat measurement of 2.44 mm CaCl_2_ titrated into 86 μm SUPC2 Ccore protein and 255 μm EDTA in pH 7.4, 25 mm Tris, 150 mm KCl, 1 mm TCEP buffer at 25 °C, using 32 injections of 1.49 μl/injection. *I*, simultaneously fitted, baseline-corrected isotherms of SUPC2 Ccore only (*blue trace*) and SUPC2 Ccore with EDTA (*red trace*) ITC experiment.

To determine whether there are additional Ca^2+^-binding sites outside the EF-hand region, we needed to be certain that both the binding stoichiometry and the binding affinity of the HPC Cterm were measured with a high degree of accuracy. Because Ca^2+^ is necessary for the expression and purification of the protein, it is inherently difficult to maintain a Ca^2+^-free protein sample without the use of Ca^2+^ chelators. If the *K_D_* for the Ca^2+^-binding affinity of the protein lies in a low micromolar range, even a micromolar level of residual Ca^2+^ in the sample will render a significant proportion of protein unavailable for ITC characterization. To account for the residual Ca^2+^ bound to the protein, two ITC experiments were set up to pair each “Ca^2+^-HPC2Cterm” experiment (Fig. 3*A*) with a matching “Ca^2+^-HPC2Cterm + chelator” experiment (Fig. 3*B*). The thermodynamic parameters of the binding interaction were determined by fitting both binding isotherms simultaneously to an identical binding site model (Fig. 3*C*). The presence of the Ca^2+^ chelator enabled us for the first time to definitively determine the binding stoichiometry, *N*, by accounting for the effects of residual Ca^2+^. The best fit values for binding stoichiometry are very close to 1 (Table 2), indicating that there is only one Ca^2+^-binding site, presumed to be the same Ca^2+^-binding site previously identified in the HPC2 C-EF. The best fit value for the binding affinity *K_D_* is 22 μm. Compared with the known HPC2 C-EF binding affinity (*K_d_* ∼461 μm) (17), the Ca^2+^-binding affinity is significantly enhanced (20-fold increase) in the longer and trimeric HPC2 Cterm construct.

**TABLE 2:** **Binding parameters for HPC2 Cterm and Ca^2+^ interaction** Fitting results for thermodynamic parameters of HPC2 Cterm and Ca^2+^-binding interactions based on the *identical binding sites model*. (The binding stoichiometry is defined as the number of Ca^2+^-binding sites within each HPC2 Cterm monomer. Parentheses indicate 95% confidence intervals.)

Molar enthalpy (Δ*H*)	Dissociation constant (*K_D_*)	Binding stoichiometry (*N*)	Residual Ca^2+^ (*R_o_*)
*kcal*	μ*m*		μ*m*
−10.6 (−11.8, −9.62)	22.3 (19.2, 25.7)	0.905 (0.854, 0.950)	19.7 (14.7, 25.9)

##### SUPC2 C-EF and SUPC2 Ccore Both Contain Two Different Ca^2+^-binding Sites That Exhibit Positive Cooperativity

To determine the Ca^2+^-binding properties of SUPC2 C-EF with the presence of residual Ca^2+^, we also used a Ca^2+^ chelator in the ITC experiments (Fig. 3, *D* and *E*). The binding model that best describes the ITC data is the two cooperative binding sites model. In this model, binding of one site affects the binding affinity of the other site by a factor defined as the cooperativity coefficient (*c*). In this binding model, the best fit values for *K_D_* of the two Ca^2+^-binding sites are 6 and 19 μm, respectively, with a *c* value of 3.1, indicating positive cooperativity (Table 3). The positive cooperativity observed between the two Ca^2+^-binding sites agrees with other EF-hand proteins that contain paired Ca^2+^-binding sites (33, 34).

**TABLE 3:** **Binding parameters for SUPC2 C-EF and Ca^2+^ interaction** Fitting results for thermodynamic parameters of SUPC2 C-EF and Ca^2+^-binding interactions based on the *two cooperative binding sites model*. (The number of Ca^2+^-binding sites is specific to each SUPC2 C-EF monomer. Parentheses indicate the 95% confidence interval of parameters when *c* is fixed as 3.1.)

Site 1 molar enthalpy (Δ*H*1)	Site 1 dissociation constant (*K_D_*_1_)	Site 2 molar enthalpy (Δ*H*2)	Site 2 dissociation constant (*K_D_*_2_)	Cooperativity coefficient	Residual Ca^2+^ (*R_o_*)
*kcal*	μ*m*	*kcal*	μ*m*		μ*m*
−16.7 (−20.0, −13.9)	6.03 (4.91, 7.21)	−3.32 (−6.27, −1.49)	19.2 (14.6, 25.8)	3.10 (fixed)	28.9 (28.2, 29.8)

To identify possible additional Ca^2+^-binding sites outside the EF-hand domain, we further examined the Ca^2+^-binding properties in the longer SUPC2 Ccore construct (Fig. 3, *G* and *H*). As with the SUPC2 C-EF protein, the ITC data of the SUPC2 Ccore was best described using the two cooperative binding sites model (Fig. 3*I*). With the cooperative binding model, the best fit values for *K_D_*_1_ and *K_D_*_2_ are 7.65 and 48.6 μm, respectively, with *c* at a value of 2.55, also indicating positive cooperativity (Table 4).

**TABLE 4:** **Binding parameters for SUPC2 Ccore and Ca^2+^ interaction** Fitting results for thermodynamic parameters of SUPC2 Ccore and Ca^2+^-binding interactions based on the *two cooperative binding sites model*. (The number of Ca^2+^-binding sites is specific to each SUPC2 Ccore monomer. Parentheses indicate the 95% confidence interval of parameters when *c* is fixed as 2.55.)

Site 1 molar enthalpy (Δ*H*1)	Site 1 dissociation constant (*K_D_*_1_)	Site 2 molar enthalpy (Δ*H*2)	Site 2 dissociation constant (*K_D_*_2_)	Cooperativity coefficient	Residual Ca^2+^ (*R_o_*)
*kcal*	μ*m*	*kcal*	μ*m*		μ*m*
−14.7 (−22.0, −10.0)	7.65 (5.24, 13.2)	1.28 (−0.867, 6.06)	48.6 (40.0, 87.2)	2.55 (fixed)	76.9 (49.5, 90.5)

The ITC binding results provide the first direct biophysical evidence for the binding interaction between Ca^2+^ and the SUPC2 C-EF, supporting the two intact Ca^2+^-binding loops observed in the NMR structure (35). The ITC results also confirm that the SUPC2 C-EF binds Ca^2+^ more tightly compared with HPC2 C-EF. The ITC results with the SUPC2 C-EF and the SUPC2 Ccore also support the finding that both protein constructs contain the same number of Ca^2+^-binding sites, with similar Ca^2+^-binding affinities and cooperative behavior. Similarly, these results again indicate that there are no additional Ca^2+^-binding sites in the longer SUPC2 Ccore protein outside the EF-hand region. Therefore, although the HPC2 Cterm and SUPC2 Ccore contain different numbers of Ca^2+^-binding sites, those Ca^2+^-binding sites are only located in the EF-hand region.

##### NMR Characterization Revealed the Two Ca^2+^-binding Sites in the SUPC2 Ccore Construct

**FIGURE 4.**
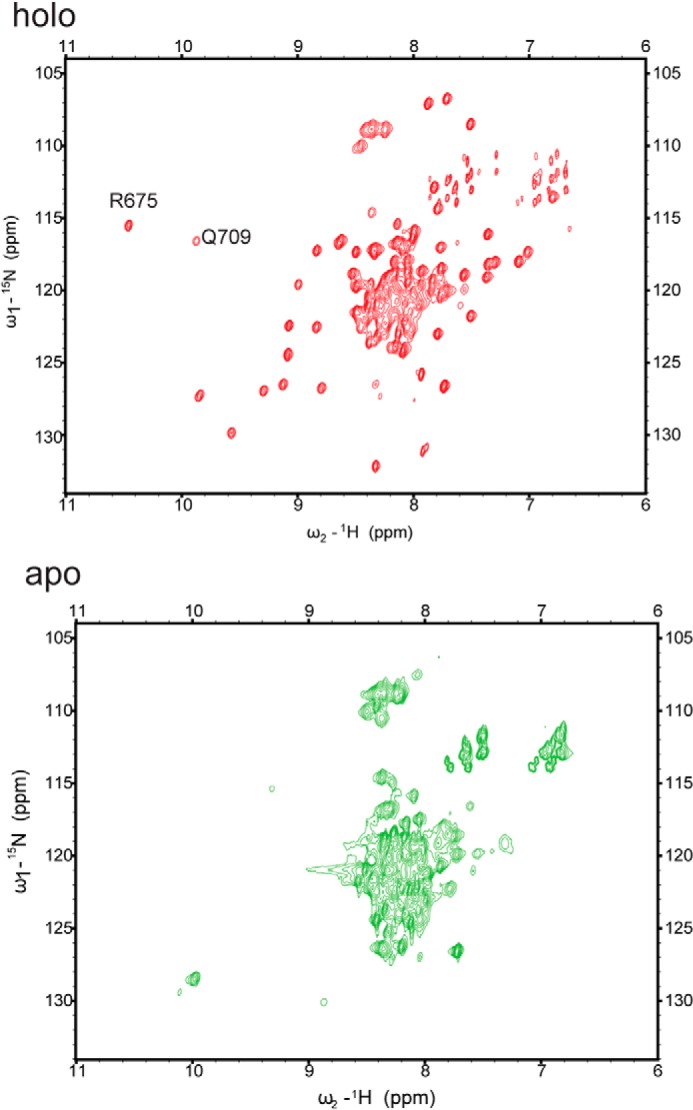
**NMR spectra of SUPC2 Ccore under holo and apo conditions.** Comparison of ^1^H-^15^N HSQC NMR spectra of ^13^C^15^N SUPC2 Ccore in the Ca^2+^-saturating condition (red contours) and in the Ca^2+^-free condition (green contours) in pH 7.4, 2 mm Tris-d_11_, 150 mm KCl buffer with 1 mm TCEP at 25 °C.

To map the Ca^2+^-binding elements in the SUPC2 Ccore structure, two-dimensional HSQC and a series of three-dimensional NMR experiments were collected with this protein under both holo and apo conditions. In the spectra collected under holo conditions, the majority of the peaks were well resolved and dispersed with different chemical shifts, clearly indicating that the SUPC2 Ccore sample is well folded under saturating Ca^2+^ conditions (Fig. 4*A*). The majority of the well resolved peaks were assigned based on three-dimensional ^13^C-^15^N-^1^H experiment spectra in SUPC2 Ccore. Nevertheless, there were an insufficient number of peaks in the three-dimensional NMR spectra to account for all the residues in the SUPC2 Ccore protein. For instance, there are only 145 peaks in the HNCO spectrum, whereas there are 192 residues in the SUPC2 Ccore construct. After residue assignment, we found that most of the assigned peaks belong to residues in the EF-hand and L2 regions of the SUPC2 Ccore protein. The peaks that belong to residues in the downstream coiled-coil region are not present in these spectra, possibly due to the line broadening from a combination of slow rotation rate and conformational exchange. In the ^1^H-^15^N HSQC spectra recorded under holo conditions, the peaks corresponding to the residues in the EF-hand region appear to have much narrower line widths than what would be expected for a trimeric protein of 64 kDa. Instead, the line widths of these peaks are comparable with that for the monomeric SUPC2 C-EF protein, suggesting that each EF-hand domain moves on a faster time scale compared with the rest of the protein.

The NMR spectra under the holo state also indicate that there are two Ca^2+^-binding sites in the SUPC2 Ccore protein. In the ^1^H-^15^N HSQC spectra, two peaks (Q675 and R709) appeared in the downfield ^1^H chemical shift region. These peaks correspond to residues located in the two Ca^2+^-binding sites (Fig. 4*A*), because their amide protons are involved in hydrogen bonding with the carboxyl oxygen atom of the corresponding aspartate side chain in the individual α-helix-loop-helix motif. In the spectra collected in the absence of Ca^2+^, the majority of the peaks in the NMR spectra are centered together, suggesting that the most of the protein, specifically the EF-hand region, is in a largely unstructured state (Fig. 4*B*). This NMR observation is consistent with the shift in *r_h_* of the SUPC2 Ccore in its apo and holo states. Because the removal of Ca^2+^ results in a largely unstructured protein, the *r_h_* of the SUPC2 Ccore seems to be larger in the apo state relative to the holo state.

##### The SUPC2 Ccore EF-hand Domain Shares Similar NMR Chemical Shifts with the SUPC2 C-EF Protein

**FIGURE 5.**
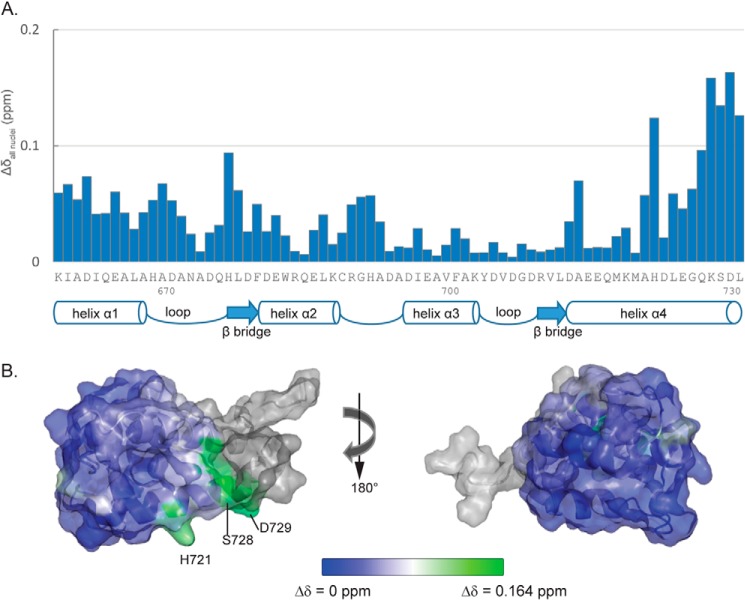
**Backbone chemical shift comparison of SUPC2 Ccore and SUPC2 C-EF in the EF-hand domain.** *A*, averaged chemical shift change of each residue between SUPC2 C-EF and SUPC2 Ccore based on five sets of nuclei. Averaged chemical shift change for each residue is calculated as stated in Equation 1. Regions of secondary structure are indicated *below* with *block arrows* representing β bridges and *cylinders* representing helical regions. *B*, an overlay of the results from the backbone chemical shifts changes on the NMR structure of the SUPC2 C-EF. Greater chemical shift changes are displayed as increasing *green intensity*. Lower chemical shift changes are displayed as increasing *blue intensity. Light gray* indicates residues for which chemical shift data are not available. The figure at the *right* is rotated 180° around the *y* axis.

We mapped the chemical shift perturbations to determine whether there are any domain-domain interactions within the trimeric SUPC2 Ccore construct. We calculated the perturbations by comparing the chemical shifts of assigned backbone atoms in the SUPC2 C-EF and the SUPC2 Ccore under holo conditions. We found that the majority of the chemical shifts in the EF-hand domain remain unchanged in the longer and trimeric SUPC2 Ccore protein. Most importantly, the two paired α helix-loop-α helix structural motifs that form the EF-hand region remain undisrupted (Fig. 5). The largest changes in chemical shifts, still less than 0.2 ppm, were detected near the N and C termini of the EF-hand domain. These changes are consistent with a decreased helicity in these residues in the SUPC2 Ccore construct compared with the SUPC2 C-EF (36), most likely due to the designed mutations at the end of the helices to achieve protease resistance. The perturbed residues are involved in the interaction of helices α1 and α4. Such effects could explain the slightly lower Ca^2+^-binding affinity of the SUPC2 Ccore (Tables 3 and 4). However, because the majority of the EF-hand domain remains the same, the ITC results also indicate that the two protein constructs share similar Ca^2+^-binding profiles. Hence, we report that there are few structural differences between the EF-hand regions of the two constructs. Our results suggest that there are no interactions between the EF-hand and coiled-coil/L2 linker in the SU Ccore protein or among different EF-hand domains in the SUPC2 Ccore trimer complex.

## DISCUSSION

The C-terminal tail of PC2 has been shown to be responsible for the assembly and function of the PC2 channel. However, the oligomeric states and Ca^2+^-binding properties of the PC2 C-terminal tail have not been fully elucidated previously because the HPC2 Cterm is prone to proteolytic degradation and aggregation in solution without the presence of Ca^2+^. We overcame the technical barriers of studying the HPC2 Cterm by establishing new solution conditions to prevent aggregation and have designed a more stable SUPC2 Ccore construct, which is resistant to proteolytic degradation. We have also applied a new approach to analyze ITC results that accounts for the residual Ca^2+^ in the ITC sample. With the technical advances, we have determined that the C-terminal tail of PC2 forms trimers in solution with and without Ca^2+^, and the trimer is independent of the protein concentration in the examined range. Using ITC, we have defined the Ca^2+^-binding profiles in the trimeric HPC2 Cterm and demonstrate that there are no additional Ca^2+^-binding sites outside of its EF-hand region. However, the Ca^2+^-binding affinity of the HPC2 Cterm is much higher compared with the monomeric HPC2 C-EF. In sea urchin PC2, we also established that both the SUPC2 C-EF and the Ccore have the same number of Ca^2+^-binding sites. In contrast to the human orthologues, the SUPC2 C-EF and the Ccore share similar Ca^2+^-binding profiles, both containing two cooperative binding sites with similar affinities. The NMR characterization of the SUPC2 Ccore in its holo state shows that the EF-hand region rotates freely and does not interact with the coiled-coil domain or with other EF-hand domains.

### 

#### 

##### The Coiled-coil Domain within HPC2 Cterm and SUPC2 Ccore Determines the Trimeric State

As a member of the TRP channel family, the PC2 channel is generally believed to be a homotetramer, and its C-terminal domain is essential for channel assembly. However, the x-ray structure of the isolated coiled-coil domain of human PC2 is a trimer. We investigated the oligomeric state of HPC2 Cterm and SUPC2 Ccore in solution to determine whether it is consistent with the tetrameric channel assembly or the trimeric coiled-coil crystal structure. Using SEC-MALS analysis, we determined that both the HPC2 Cterm and the SUPC2 Ccore form trimers in solution. Moreover, our results indicate that the trimer formation is independent of both protein and Ca^2+^ concentrations, whereas the isolated HPC2 C-EF and SUPC2 C-EF have been previously shown to be monomeric in solution in both apo and holo states (35). This trimer formation of HPC2 Cterm and SUPC2 Ccore in solution contradicts the generally believed tetrameric assembly of TRP channels. However, it is important to note that there are other structural motifs shown to be essential for channel assembly in addition to the C-terminal domain (11, 37). We believe the channel assembly is determined by both the transmembrane domains and the cytosolic domains. In solution, the trimer complex is the preferred form, because it is primarily driven by hydrophobic interactions throughout the coiled-coil domain. However, in the context of the full-length PC2 sequence and with the presence of the transmembrane domains, the C-terminal tail of PC2 could form a tetramer. Thus, in the tetramer, the coiled-coil trimer can interact with an additional α-helix from an adjacent PC2 molecule when they are brought together via the transmembrane domains in the lipid membrane, where the effective concentration of proteins are greater. Therefore, the trimer formation could be a necessary intermediate to tetramer formation. These non-conventional tetramers generated from an α-helix monomer and a coiled-coil trimer have been observed in several known protein structures (38).

##### HPC2 Cterm Contains One Ca^2+^-binding Site and Binds Ca^2+^ with Significantly Higher Affinity Compared with the Isolated HPC2 C-EF

Previous studies of HPC2 C-EF have demonstrated the existence of one weak Ca^2+^-binding site (*K_D_* ∼461 μm), an affinity that is not coincident with the physiologic range of Ca^2+^ concentrations in the cytoplasm (17). Our ITC analysis shows that the HPC2 Cterm contains the same number of Ca^2+^-binding sites as HPC2 C-EF. However, the affinity of the same binding site differs drastically in HPC2 Cterm (*K_D_* ∼22 μm, 20-fold increase). The improved binding affinity suggests that the trimeric composition is the key to enhancing the intrinsically weak Ca^2+^-binding site identified in the EF-hand domain. Such improvement of binding affinity could result from a combination of 1) stabilizing interactions between EF-hand and other regions of the HPC2 Cterm, 2) positive interactions among different EF-hand domains within the same trimer, and 3) the restraints of movement imposed on the EF-hand domain by the trimeric coiled-coil region. Because the Ca^2+^-binding affinity of HPC2 C-EF is intrinsically low, these interactions could provide significant stabilizing effects in the EF-hand domain, which translate to the greatly enhanced binding affinity.

The improved Ca^2+^-binding affinity of HPC2 Cterm is in the physiologically relevant Ca^2+^ range for PC2 channel deactivation. However, this increased affinity does not explain how the channel is activated by cytoplasmic Ca^2+^ concentrations near 100 nm. However, it is possible that additional factors present within the complete channel expressed in eukaryotic cells may contribute a higher sensitivity to submicromolar Ca^2+^ concentrations. These could include higher order structural interactions within the fully tetrameric channel or post-translational modifications. Indeed, several post-translational sites located in the PC2 C-terminal L2 region are known to change the Ca^2+^-dependent response of the PC2 channel (5, 9, 39). Because the HPC2 Cterm in our study was expressed using recombinant methods, our sample does not have the essential post-translational modifications required for sensing Ca^2+^ level changes *in vivo*. In addition, the N-terminal tail and other intracellular loops of PC2 are also involved in channel regulation (6, 37). It is equally possible that these regions contain other Ca^2+^-binding elements that are involved in the activation of the channel. It is also possible that there could be increased Ca^2+^-binding affinity in the tetrameric assembly of the full-length sequence as proposed above.

##### SUPC2 C-EF and SUPC2 Ccore Bind to Ca^2+^ with the Same Stoichiometry and Similar Affinities

The NMR and ITC results both suggest that there are two Ca^2+^-binding sites in the SUPC2 C-EF and the SUPC2 Ccore proteins. The NMR results also suggest that Ca^2+^-binding is necessary to stabilize the protein. The presence of the same number of binding sites shows that there are no additional Ca^2+^-binding sites outside the EF-hand region, similar to the results seen in the human PC2. Different from human PC2, the two constructs share similar Ca^2+^-binding affinities (Tables 3 and 4). Such differences in their Ca^2+^-binding properties indicate that the human and sea urchin have different Ca^2+^-binding mechanisms. Both the HPC2 C-EF and the SUPC2 C-EF contain a pair of α-helix-loop-helix motifs. However, prior NMR studies have revealed that the first Ca^2+^ binding loop in HPC2 C-EF is truncated (9), rendering the first α-helix-loop-helix motif non-functional, whereas both binding loops remain intact in SUPC2 C-EF (35). In the EF-hand region of sea urchin, the two Ca^2+^-binding loops can interact with each other by forming anti-parallel β-sheet and stabilize the structural integrity of the four-helix bundle (35). Consequently, the SUPC2 C-EF binds to Ca^2+^ with much stronger affinity, relative to HPC2 C-EF. As their similar Ca^2+^-binding profiles suggest, the SUPC2 Ccore trimer does not provide further stabilizing effects to the inherently stable SUPC2 C-EF. In addition, the NMR analysis shows that the EF-hand region does not interact with another EF-hand or the coiled-coil region in the SUPC2 Ccore construct. Therefore, we believe that the trimeric formation of SUPC2 Ccore does not affect how its EF-hand region binds to Ca^2+^.

The molecular motion of the PC2 C-terminal domain trimer complex can be best described as a three-headed flail model. The elongated coiled-coil domain keeps the trimer together like the handle of the flail. The L2 region connecting the coiled-coil region and the EF-hand region provides the structural flexibility for the trimer complex like the flail chains. Each EF-hand domain in the trimer complex can move independent of each other like the three heads of the flail. In sea urchin PC2, each EF-hand domain is stable and rigid; therefore, the EF-hand can still move and bind to Ca^2+^ as monomers. In human PC2, the loss of one Ca^2+^-binding site is associated with structural destabilization that cannot be overcome with Ca^2+^ binding to the isolated EF hand. Instead, we hypothesize that in the full-length HPC2 Cterm, the EF-hands are linked together through the coiled-coil domains as trimers, providing structural stabilization. The differences observed between human and sea urchin C-terminal domains are another example of the protein evolutionary process, in which some degree of structural stability of the protein was lost and resulted in possible free energy difference for cooperative and regulatory effects.

In conclusion, our results indicate that both human and sea urchin PC2 C-terminal tails form trimers in solution. However, the trimerization has different effects on their Ca^2+^-binding properties in human and sea urchin PC2 C-terminal domains. In human PC2, the trimer formation is important to enhance the Ca^2+^-binding affinity of the intrinsically weak binding site, allowing the *K_D_* of HPC2 Cterm to be in the physiological range for channel regulation. In contrast, the SUPC2 C-terminal domain trimer does not further improve its binding affinity from its intrinsic binding affinity. Although the study is focused on the solution properties of PC2 C-terminal domains, it also provides biophysical insights into how the C-terminal domain could behave in the full-length channel form. Our study not only expands the understanding of how PC2 is regulated by cellular Ca^2+^ level changes but also provides a useful strategy in characterizing other Ca^2+^-binding proteins.

## Footnotes

5 The abbreviations used are:

PC2: polycystin-2
HPC2: human polycystin-2
HPC2 Cterm: human polycystin-2 C-terminal construct, which includes the full-length cytoplasmic tail
HPC2 C-EF: human polycystin-2 C-terminal EF-hand construct, which contains the isolated EF-hand domain
SUPC2: sea urchin polycystin-2
SUPC2 Ccore: sea urchin polycystin-2 C-terminal construct, which includes the EF-hand, linker 2, and coiled-coil domains
SUPC2 C-EF: sea urchin polycystin-2 C-terminal EF-hand construct, which contains the isolated EF-hand domain
TRP: transient receptor potential
ITC: isothermal titration calorimetry
TCEP: tris(2-carboxyethyl)phosphine
SEC: size exclusion chromatography
MALS: multiangle light scattering
HSQC: heteronuclear single quantum coherence
*r_h_*: hydrodynamic radius
BAPTA: 1,2-bis(2-aminophenoxy)ethane-*N*,*N*,*N*′,*N*′-tetraacetic acid.
